# Seven insights from Albert Camus’s Plague about epidemics, public health and morality

**DOI:** 10.1093/pubmed/fdae267

**Published:** 2024-10-02

**Authors:** Steven R Kraaijeveld

**Affiliations:** Department of Ethics, Law & Medical Humanities, Amsterdam University Medical Center, The Netherlands

**Keywords:** epidemiology, ethics, public health, epidemics, Camus

## Abstract

For Albert Camus, plague was both a fact of life and a powerful metaphor for the human condition. Camus engaged most explicitly and extensively with the subject of plague in his 1947 novel, *The Plague* (*La peste*), which chronicles an outbreak of what is presumably cholera in the French-Algerian city of Oran. I often thought of this novel—and what it might teach us—during the recent COVID-19 pandemic. In this article, I discuss seven important insights from *The Plague* about epidemics, public health and morality.

The French writer, playwright and philosopher Albert Camus was deeply fascinated with plague both as a fact of life and as a metaphor for human existence. Camus wrote about plague in various places throughout his oeuvre. His 1948 play *State of Siege* (*L’État de siège*), for instance, includes a personification of plague in the form of a character—The Plague—who torments the slumbering Spanish city of Cadiz. It is his 1947 novel *The Plague* (*La peste*), however, which offers Camus’s most wide-ranging treatment of the subject.

*The Plague* is set in the French-Algerian city of Oran in the 1940s, where a contagious disease (presumably cholera) has broken out. A narrator—whose identity is not revealed until the end of the novel—chronicles the tale of how the inhabitants of Oran and several outsiders face, struggle through and work together against the plague. The novel thus falls within a centuries-long tradition of representing disease outbreaks through the medium of art.^1^

At least two historical sources for *The Plague* can be distinguished: the cholera epidemic that decimated Oran’s population in 1849, and the world wars that ravaged Europe and beyond in the first half of the 20th century.^2^ Although the concept of plague clearly has political significance for Camus (e.g. as a metaphor for Nazism), I will not consider this aspect here. Instead, I will remain close to the medical meaning of plague.

As a philosopher and public health ethicist, Camus’s novel often came to mind during the recent coronavirus disease 2019 (COVID-19) pandemic. In what follows, I discuss seven important insights from The Plague about epidemics, public health and morality.

The first insight is that plague is an inherent and recurring part of life. One should not think of the plague as existing outside of the human realm and only occasionally entering our lives and societies, as if from some remote and disconnected place. Plague is just as much a part of life as health or peace. It belongs to our very form of life: living closely together in groups. As the eccentric Cottard remarks to Dr Rieux: ‘All those folks are saying, “It was plague. We’ve had the plague here.” […] But what does that mean—“plague”? Just life…’^3^^(p270)^ Plague consequently cannot be *overcome* in any complete or definitive sense. As Rieux concludes in the novel’s final passages, when the town’s long-shut gates—closing off the ailing city from the world—have reopened at last: there is no ‘final victory’ and joy is ‘always imperiled’.^3^^(p271)^ This is not an affirmation of pessimism or despair, but rather an invitation to anyone ‘refusing to bow down to pestilences’^3^^(p271)^ to remain attentive and responsive.

The second insight is that plague is always unexpected. This may seem counterintuitive or even contradictory to the previous insight. If plagues are a persistent part of life, then why would they be unexpected? There are at least two reasons. First, despite their commonalities, plagues are never quite the same. They take different forms; outbreaks of infectious diseases do not lend themselves to being predicted or modeled with absolute certainty. The epidemiological circumstances of outbreaks—transmission dynamics, disease progression, and so on—will always contain elements of uncertainty, even if the eventual reemergence of plague can be taken as a brute fact. Large outbreaks tend to be highly disruptive of social life. Such disruption, especially when it occurs across a wide range of social domains (e.g. family life, work, education, etc.), adds to the unpredictability of the effects of plague and complicates the possibility of anything like full preparedness. Second, and more importantly, the intellectual conviction that plagues will reoccur can never fully prepare us for their sheer *reality*: for their myriad psychological, emotional, social and existential effects. Theoretical knowledge and lived experience never fully align: ‘Everybody knows that pestilences have a way of recurring in the world; yet somehow we find it hard to believe in ones that crash down on our heads from a blue sky.’^3^^(p35)^ Yes, plagues happen: but why should they happen to us? And why now? The shock of the reality of disease outbreaks—a concrete confrontation with plague—will always contain psychological elements of surprise and disbelief.

The third insight is that plague will take its course unless it is checked by the human will. Plague will spread far and wide. It is the nature of infectious diseases to do so. Curbing the course that an outbreak will take—and thereby limiting its impact on individuals and societies—requires willpower. This idea leads to one of the most powerful statements in the novel, made by the wanderer Tarrou: ‘What’s natural is the microbe. All the rest—health, integrity, purity (if you like)—is a product of the human will, of a vigilance that must never falter.’^3^^(p224)^ Plague, disease, illness, the microbe: this is the natural order. These are not aberrations, occasional interruptions to a steady baseline of robust human health. Yet, we can—and must—contest this natural order of the microbe through active and continued opposition. Wellbeing is not a given; it must be forged.

The fourth insight is that plague discloses and challenges our morality. Hypothetically, in a time of complete health and peace—in the perfect absence of any need for anyone to struggle in the world—there is little scope for morality. It is precisely when we are forced to face a phenomenon like plague in all its grim reality that our morality is tested. As Tarrou tells Dr Rieux: ‘The good man, the man who infects hardly anyone, is the man who has the fewest lapses of attention. And it needs tremendous will-power, a never-ending tension of the mind, to avoid such lapses.’^3^^(p224)^ Morality requires careful attention—constant vigilance. This is a recurring theme in Camus’s work. To confront plague, such attentiveness is required at both a collective and individual level. This can be exhausting; as Tarrou laments, this is why ‘those who want to get the plague out of their systems, feel such desperate weariness.’^3^^(p224)^ Although it can be tempting to give in, one must not give up. The decision by the journalist Rambert to stay in Oran to help fight the plague, despite being offered a way out of the city by smugglers, discloses his morality and is an example to others.

The fifth insight is that we should not give too much importance to exceptional moral behavior. Facing plague is about solidarity. It requires collective effort and cooperation; we must all do our part. If we all do our part, then no single person has to be a moral saint. There is no need, then, to applaud exceptional individual moral behavior. In fact, we should celebrate the many smaller—but good—parts that people play in times of plague. On this point, it is worth quoting Camus at length:

‘[B]y attributing overimportance to praiseworthy action one may, by implication, be paying indirect but potent homage to the worst side of human nature. For this attitude implies that such actions shine out as rare exceptions, while callousness and apathy are the general rule. The narrator does not share that view.’^3^^(p115)^

Presumably, focusing on the worst side of human nature or particularly bad individual behavior will have the same effect. There are few instances in *The Plague* of explicit judgment, condemnation and moralization of the behavior of others (cf. the COVID-19 pandemic).^4^ This is a call to dogged optimism: let us emphasize the mundane but robust goodness of the larger part of humanity. This means that governments ought to eschew sanctifying or vilifying its citizens, which may be difficult when such moralization is desired or even insisted upon by (part of) the public, or when relying on people’s genuine solidarity is or appears to be insufficient to achieve infectious disease control (which, in itself, may fuel political criticism).

The sixth insight is that perfectionism is an enemy to progress. This is exemplified by one of my favorite characters in The Plague—a humble clerk by the name of Joseph Grand. Grand tracks the plague’s death rate and other data for Oran’s Municipal Office. He has been writing a book for a long time, but he is unable to get past the first sentence. He assures us (and reassures himself): ‘Once I’ve succeeded in rendering perfectly the picture in my mind’s eye […] the rest will come more easily.’^3^^(94)^ But the rest never follows. There are many ways to interpret Grand’s predicament. One way to do so, I want to suggest here, is that the attempt to perfect a response to a challenging task—i.e. to facing plague—is often detrimental. There is no ideal start. One will never be perfectly in control. The most important thing to do is to conceive of a good plan and to execute it well. There will be hurdles and knocks along the way; we must not only allow for this, but we must resist letting inevitable imperfections freeze us like Grand’s opening sentence.

Finally, the seventh insight is that the presence or absence of plague is a contentious matter. People will disagree that plague has come or gone—even in the face of strong evidence to the contrary. After the concierge of his building falls ill and dies of a suspicious fever, Rieux consults a fellow doctor by the name of Castel. Rieux and Castel quickly realize that the concierge’s illness was likely due to plague. They subsequently try to warn the authorities, to no avail. ‘All that could be said at present was that we had to deal with a special type of fever; […] it was unwise to jump to conclusions.’^3^^(p45–46)^ Castel observes that the authorities know quite well that it is plague, but that they are reluctant to acknowledge it, because ‘were this to be officially admitted, the authorities would be compelled to take very drastic steps.’^3^^(46)^ It is only when the number of deaths in the city spikes, panic spreads and newspapers are forced to report on the events that denial is no longer an option. And if it can be difficult to accept the arrival of plague, it can also be hard to let it go after one has lived with it. This is exemplified by a powerful scene at the end the novel. After the plague has left Oran—symbolized by the ceremonial reopening of the city’s gates—Dr Rieux is halted on the street by a police cordon. ‘There’s a crazy fellow with a gun, shooting at everybody,’^3^^(p266)^ a policeman tells the doctor. The fellow turns out to be Cottard; one of the characters who has managed to thrive during the plague (for reasons too complex to address here). Having felt an increasing sense of consternation and disbelief at the retreat of the plague, and refusing to return to a life without it, Cottard has gone mad.

I have selected these seven insights from *The Plague*. There are bound to be more. I invite everyone—especially those involved in public health—to read or reread the novel and to further reflect on its lessons.

What Camus drives home is that, for as long as there is life, there will be plague. And what he portrays so memorably is that, for as long as there are human beings, there will be resistance to disease. This resistance can take many forms. A doctor may dedicate her life to treating patients. An epidemiologist might devote his life to advancing good public health. Or an artist like Camus can provide us with existential lessons in the form of allegories.

In the end, when confronted by a contagious disease, most people will simply try to survive and live as best they can. This is, to use a phrase from Camus’s notebooks, the ‘unshadowed light of tragic and mortal things.’^5^^(p135)^
